# Kinetic and Thermodynamic Analysis of Algal Lipid Extraction and Physicochemical Characterization for Biodiesel Production

**DOI:** 10.1021/acsomega.6c01770

**Published:** 2026-06-18

**Authors:** Mohammad Aliff Shakir, Chen Xu, Wardah Senusi, Mardiana Idayu Ahmad

**Affiliations:** † School of Industrial Technology, 26689 Universiti Sains Malaysia, Minden Heights, Penang 11800, Malaysia; ‡ Renewable Biomass Transformation Cluster, School of Industrial Technology, Universiti Sains Malaysia, Minden Heights, Penang 11800, Malaysia

## Abstract

Marine macroalgae such as *Ulva lactuca* are a rich source of lipid, offering a renewable, nonedible biomass for clean energy conversion. These biological macromolecules exhibit structural diversity and functional properties that make them suitable feedstocks for biodiesel production. However, efficient utilization is hindered by a limited understanding of their extraction kinetics and thermodynamic behavior under process conditions. This study investigates the apparent extraction kinetics and thermodynamic behavior of lipids from *Ulva lactuca* using Soxhlet extraction, while evaluating their potential for biodiesel production. Soxhlet extraction was conducted using a methanol-hexane solvent system at varying temperatures (75–95 °C) and durations (60–360 min) for both ground (50–250 μm) and unground (>250 μm) samples. Results revealed that ground biomass exhibited higher lipid yields (5.04%) compared to unground biomass (4.06%). The extraction process followed a second-order kinetic model, indicating intraparticle diffusion behavior. Thermodynamic analysis indicated that the extraction process is endothermic and nonspontaneous, reflecting its strong dependence on temperature. FTIR analysis confirmed the presence of lipid-based functional groups in the extract. Physicochemical characterization showed an acid value of 19.16 mg KOH/g, an iodine value of 56.01 g I_2_/100 g, and a kinematic viscosity of 12.1 mm^2^ /s. These findings highlight the potential of *Ulva lactuca* lipids as a renewable resource for biodiesel production, although further pretreatment is required to meet fuel quality standards.

## Graphical abstract

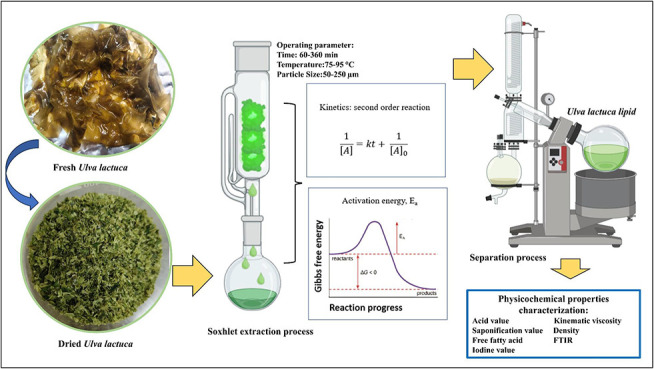

## 1 Introduction

The increasing demand for cleaner energy has driven interest in renewable biological resources, particularly lipids, as sustainable alternatives to fossil fuels. Marine algae represent a promising nonedible source for biodiesel due to their renewability, high growth rate, and low environmental impact, supporting the global transition toward sustainable energy systems.^1^ Biodiesel is a renewable and clean fuel that has gained attention for its eco-friendly properties and diverse feedstocks, including vegetable oils, animal fats, and waste oils. These feedstocks significantly lower carbon emissions and reduce pollution.^2^ Among these, lipids derived from nonedible and underutilized biomass sources, such as marine algae, are increasingly recognized for their potential as sustainable macromolecular feedstocks. The use of edible feedstocks in first-generation biodiesel raises concerns regarding food-energy competition.^3^ In this context, marine macroalgae have emerged as sustainable alternatives for biodiesel production due to their nonedible nature and renewable biomass characteristics. Macroalgae contain structurally diverse lipids that are suitable for biofuel conversion, but their extraction efficiency and molecular behavior under processing conditions remain underexplored.

Marine macroalgae such as *Ulva lactuca* are considered ideal feedstocks for third-generation biodiesel due to their rapid growth, high lipid potential, and excellent carbon capture. Unlike terrestrial biomass, *Ulva lactuca* can be cultivated in saline marine environments without requiring arable land or freshwater resources.^4^ Despite its potential, third-generation biodiesel production using *Ulva lactuca* still faces technical challenges. Studies have assessed the physicochemical properties of biodiesel products from *Ulva lactuca* lipids after transesterification, confirming compliance with international fuel standards.^5^ However, research on the physicochemical characteristics of lipids preliminarily extracted from *U. lactuca* remains limited.

While the conventional Soxhlet extraction method is widely used, it is highly sensitive to parameters such as temperature, time, and particle size.^6,7^ These parameters may affect the lipid recovery efficiency and extraction behavior in macroalgal biomass systems. Numerous studies have been conducted to describe the kinetic and thermodynamic behaviors of lipid extraction processes using the Soxhlet method, providing insights into apparent mass transfer and diffusion-controlled extraction behavior. However, the impact of variables and their interactions on the kinetic and thermodynamic behaviors remains insufficiently understood.

This study aims to systematically investigate the extraction kinetics of lipids from *U. lactuca* using Soxhlet extraction by applying a second-order kinetic model, Arrhenius analysis for activation energy determination, and thermodynamic analysis based on the Eyring equation. The study focuses on evaluating the effects of extraction parameters, including temperature and particle size, on lipid yield and extraction efficiency. In addition, the extracted lipids are characterized to assess their suitability for biodiesel production in accordance with international standards (ASTM D6751 and EN 14214), thereby linking the extraction performance with fuel application. These findings contribute to a deeper understanding of lipid extraction dynamics in marine algal biomass and demonstrate the potential of *U. lactuca* as a sustainable feedstock for efficient and scalable third-generation biodiesel production.

## 2 Materials and Methods

### 2.1 Sample Preparation

Fresh specimens of *Ulva lactuca* were naturally collected from the coastal waters of Sabah, Malaysia. To remove surface impurities and suspended particulate matter, the biomass was thoroughly washed with distilled water. It was then dried in a drying oven (Memmert UF110, Germany) at 70 °C for 48 h to ensure uniformity in sample quality for subsequent lipid extraction and physicochemical characterization. To assess the impact of particle size on lipid extraction efficiency, the dried biomass was subjected to two distinct physical preparation methods: (i) coarse cutting without further size reduction (>250 μm) and (ii) fine grinding using a laboratory grinder to produce biomass within an approximate particle size range of 50–250 μm. Therefore, this study focuses on evaluating the effect of particle size reduction on lipid extraction efficiency rather than determining an optimal or critical particle size.

### 2.2 Lipid Extraction

Lipid extraction from *Ulva lactuca* was carried out using the Soxhlet apparatus to assess the influence of extraction temperature on lipid recovery efficiency. Experiments were conducted at three temperature settings: 75 °C, 85 °C, and 95 °C. Both the coarse-cut (>250 μm) and fine-ground (50–250 μm) biomass samples were processed under identical Soxhlet extraction conditions to enable direct comparison of extraction performance. Approximately 15 g of oven-dried biomass was loaded into a cellulose Soxhlet thimble and gently compacted to ensure efficient solvent percolation. A binary solvent system comprising methanol and hexane (2:1, v/v) was prepared, with a total volume of 450 mL introduced into a round-bottom flask connected to the Soxhlet setup, as shown in Figure 1. The extraction system was heated using a temperature-controlled heating mantle to maintain the desired reflux temperature. The extraction temperature reported in this study corresponds to the heating condition of the Soxhlet system and does not directly represent the local temperature within the extraction chamber. The extraction process continued until the siphoned solvent extract appeared faint in color, indicating near-complete lipid removal.

**Figure 1:**
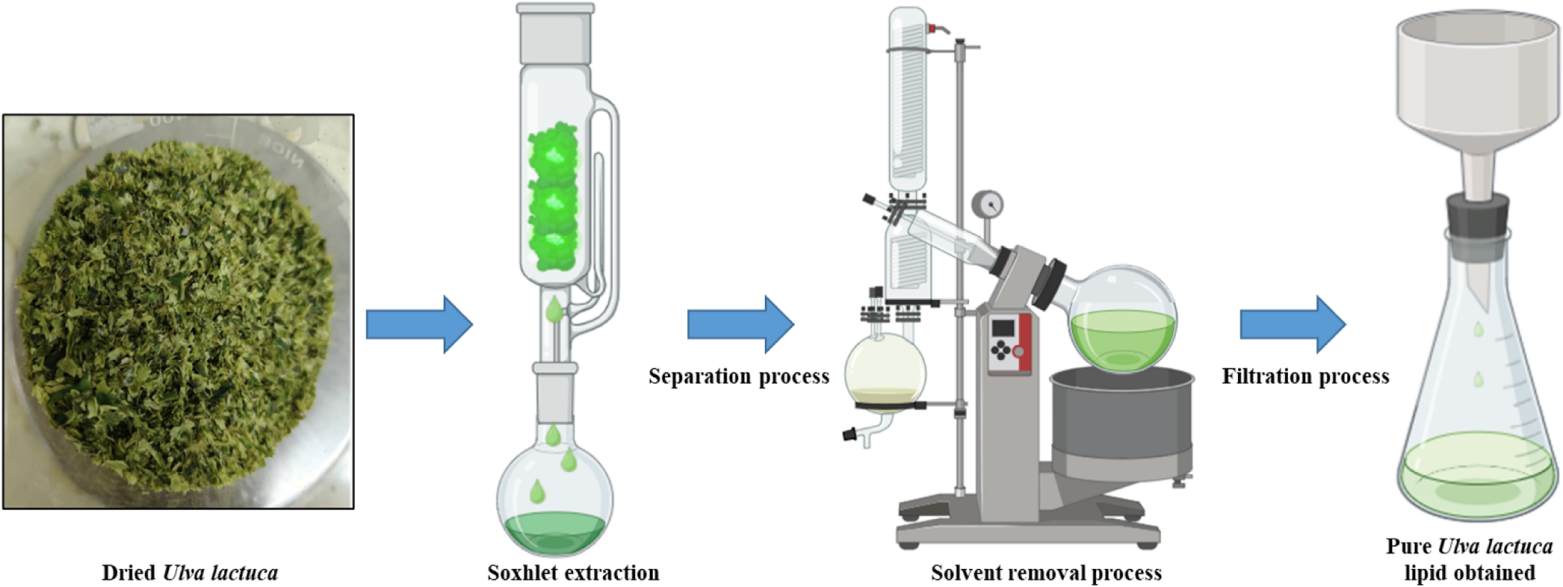
Schematic representation of the lipid extraction process from *Ulva lactuca* biomass using Soxhlet extraction.

Post-extraction, the mixture was filtered to separate residual solids, and the solvent was recovered from the lipid-rich extract using a rotary evaporator. The partially purified lipids were then dried in a hot-air oven at 65 °C for 24 h to eliminate any remaining solvent traces. Crude lipid yield was subsequently quantified gravimetrically using eq 1.

1 $$Lipid\;Yield\;(mg/g)=\frac{Weight\;of\;Extracted\;Lipids}{Weight\;of\;Dry\;Biomass}\times 100$$

### 2.3 Kinetic and Thermodynamic Modeling

The kinetic behavior of lipid extraction from *Ulva lactuca* was described by the second-order rate model, which is expressed in eq 2.

2 $$\frac{dc}{dt}=K_{S}{\;(C_{s}-C)}^{2}$$

Where *K*_S_ refers to the constant of the second-order rate in (g/mg/min), *C*_s_ refers to the equilibrium concentration of lipids extracted by Soxhlet in (mg/g), and *C* refers to the amount of lipids extracted from *Ulva lactuca* at a given time (mg/g).

Rearrangement of the second-order rate equation can be done through integration at the initial and boundary conditions *t* = 0 to *t* and *C* = 0 to *C*, which is expressed in eq 3.

3 $$\frac{t}{c}=\frac{1}{K_{s}C_{s}}+\frac{t}{C_{s}}=\frac{1}{h_{s}}+\frac{t}{C_{s}}$$

Arrhenius law was used to estimate the activation energy (*Ea* in kJ/mol) of lipids extraction from *Ulva lactuca* as expressed in eq 4.

4 $$\ln\left(K_{S}\right)=\ln\left(A\right)-\frac{E_{a}}{\mathrm{Rg}T}$$

Where *A* refers to the frequency factor in (g/mg/min), Rg refers to the gas constant, which is (8.31 J/mol/K), and *T* refers to the absolute temperature in kelvin (K).

Eyring theory was used to calculate the enthalpy (*ΔH*_a_ in kJ/mol) and the entropy (*ΔS*_a_ in kJ/mol/K) of the Soxhlet extraction process, as expressed in eq 5.

5 $$\ln\left(\frac{K_{s}}{T}\right)=-\frac{\Delta H_{a}}{RT}+\ln\left(\frac{K_{B}}{h}\right)+\frac{\Delta S_{a}}{R}$$

Where *k*_*B*_ refers to Boltzmann’s constant, which is (1.38 × 10^–23^ JK^–1^) and *h* refers to Planck’s constant, which is (6.63 × 10^–34^ Js^1–^).

Accordingly, the Gibbs free energy (*ΔG*_a_ in kJ/mol) for *Ulva lactuca*can be calculated by eq 6.

6 $$\Delta G_{a}=\Delta H_{a}-T\Delta S_{a}$$

### 2.4 Characterization of *Ulva lactuca* Lipids

*Ulva lactuca* lipid was comprehensively characterized to assess its suitability as a feedstock for biodiesel production. The physicochemical properties were evaluated using standardized analytical methods to ensure the reliability and comparability of the results. All parameters were determined in accordance with internationally recognized standards, with procedures supported by recent literature. The lipid sample used for characterization was obtained from ground biomass (50–250 μm) under the selected extraction condition of 95 °C for 6 h, corresponding to the condition that yielded the highest lipid recovery in this study.

The density of *Ulva lactuca* lipid was measured using the hydrometer method following ASTM D1298 ^8^ and procedures reported by Jayaraj.^9^ The samples were transferred into a hydrometer cylinder and placed vertically in a thermostatically controlled bath set to the required test temperature. Care was taken during sample transfer to prevent splashing, and air bubbles were removed using filter paper. After the sample temperature stabilized within ± 0.1 °C, a suitable hydrometer was gently introduced into the liquid and allowed to reach equilibrium. Density readings were recorded at the point where the liquid meniscus intersected the hydrometer scale after ensuring the absence of bubbles or irregularities on the stem.

The kinematic viscosity of *Ulva lactuca* lipid was determined using a glass capillary viscometer in accordance with ASTM D445 ^10^ and the methodology described by Aisien et al.^11^ Prior to measurement, the viscometer was thoroughly cleaned with distilled water and dried in an oven. The instrument was mounted vertically in a water bath maintained at 40 °C using a temperature-controlled hot plate, and the samples were allowed to equilibrate for 15 min. The flow time between calibration marks was recorded, and the kinematic viscosity was calculated as the average of three replicate measurements.

The acid value of the *Ulva lactuca* lipid sample was determined following ISO 660:2009,^12^ based on the procedure described by Al-Limoun.^13^ Samples weighing between 2 and 10 g were dissolved in 50 mL of ethanol and titrated with 0.1 mol L^–1^ sodium hydroxide using phenolphthalein as the indicator until a persistent pink end point was observed. The acid value (mg KOH g^–1^) and free fatty acid (FFA) content (%) were calculated using eqs 7 and 8, respectively.

7 $$AV\;(mg\;KOHg^{-1})=\frac{V\left(ml\right)\times N\left(molL^{-1}\right)\times M_{w}\left(gmol^{-1}\right)}{S_{w}\left(g\right)}$$

Where AV is the acid value (mg KOH g^–1^), *V* is the volume of potassium hydroxide used for titration (mL), *N* is the normality of sodium hydroxide (0.1 mol L^–1^), *M*_w_ is the molecular weight of sodium hydroxide (g mol^–1^) and *S*_w_ is the sample weight (g).

8 $$FFA\left(\%\right)=\frac{A_{V}\left(mgKOHg^{-1}\right)}{2}$$

Where FFA is the free fatty acid content (%) and AV is the acid value (mg KOH g^–1^).

The functional groups present in *Ulva lactuca* lipid sample were analyzed using Fourier transform infrared (FTIR) spectroscopy following the procedure reported by Kiprono et al.^14^ FTIR analyses were performed using a Nicolet iS10 spectrometer (Thermo Scientific, Waltham, USA) operating in transmission mode and an IR Prestige-21 spectrometer (Shimadzu, Kyoto, Japan) equipped with OMNIC software. Both instruments were fitted with attenuated total reflectance (ATR) accessories (ATR-8000A) to allow direct sample application. Prior to analysis, the ATR crystal was cleaned with ethanol to eliminate potential contamination. A small drop of the sample was placed directly onto the ATR crystal surface. Spectra were recorded over the wavenumber range of 4000–500 cm^–1^ with a resolution of 8 cm^–1^, a scanning speed of 20 mm s^–1^, and 32 scans per sample. Each measurement was performed in triplicate to ensure consistency and repeatability. The resulting spectra were analyzed to identify characteristic absorption bands, including C=O and C–O stretching vibrations, confirming the presence of ester functional groups indicative of lipid composition.^15^

## 3 Results and Discussion

### 3.1 Effect of Particle Size on Lipid Yield

Figure 2 shows that lipid yield increased progressively with extraction time for both particle size ranges of *Ulva lactuca* indicating that the extraction process is time-dependent and controlled by diffusion. The smaller particle size fraction (50–250 μm) produced a higher lipid yield at 5.04% compared to the coarse fraction (>250 μm) at 4.06% throughout the 360 min extraction period. This improvement is attributed to the increased surface-area-to-volume ratio of the finer particles, which enhances solvent penetration, reduces internal diffusion resistance, and promotes more efficient solvent-solid interactions.^16^ As a result, lipid dissolution occurs more rapidly, leading to a steeper increase in yield during the initial extraction stage. As extraction progresses, the rate gradually decreases due to the depletion of readily accessible lipids, eventually approaching equilibrium. The higher equilibrium yield observed for the smaller particles indicates that mechanical size reduction improves accessibility to intracellular lipids and enhances overall extraction efficiency. It should be noted that the reported lipid yield represents the total solvent-extractable fraction obtained via Soxhlet extraction, which may include not only neutral lipids but also other coextracted components such as pigments and waxes.

**Figure 2:**
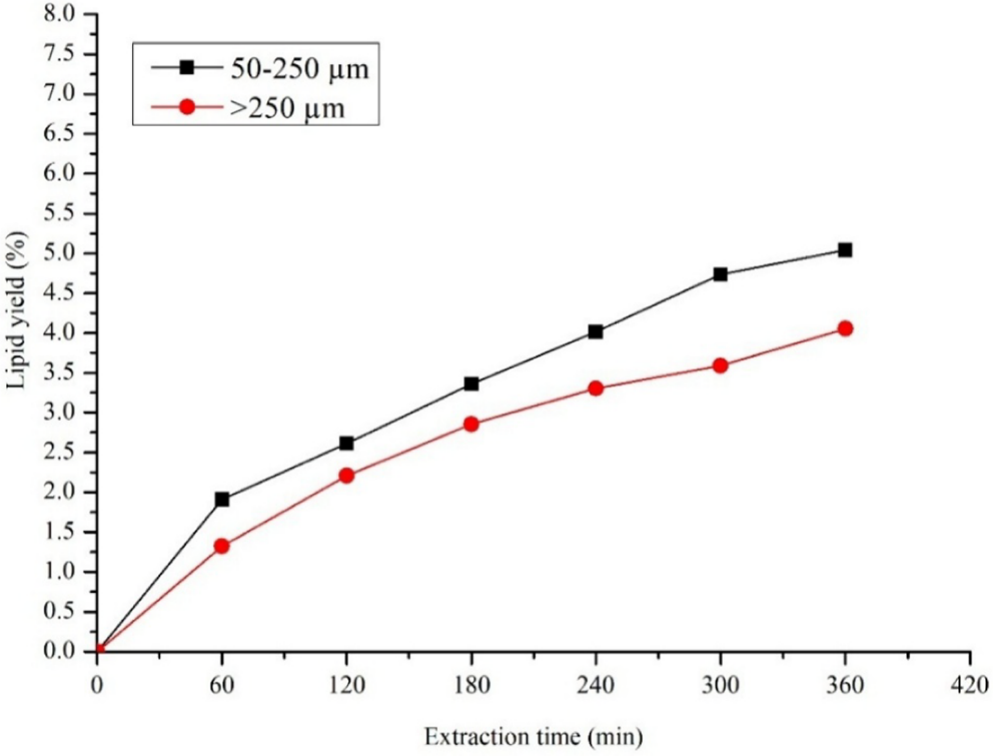
Effect of particle size on lipid yield from *Ulva lactuca* during Soxhlet extraction.

Similar improvements in lipid recovery with particle size reduction have been demonstrated in multiple studies. Aravind et al.^17^ reported that the smallest particle size of *Spirogyra* powder at 0.46 μm produced the maximum lipid yield of 26.86%, with particle size being the most influential extraction parameter. Likewise, Foerster et al.^18^ observed that reducing *Chlorella vulgaris* biomass to fine particles through ball milling increased Soxhlet-extractable lipid yield 5-fold, confirming that mechanical comminution greatly enhances solvent diffusion and lipid solubilization.

The plateau region observed at longer extraction times for both particle size groups suggests that most extractable lipids were already removed, leaving only tightly bound or less soluble components. This behavior is typical of intraparticle diffusion systems, in which the driving force for mass transfer diminishes with time. The smaller particle fraction achieved a higher equilibrium yield, confirming that the disruption of the algal matrix is critical for releasing bound lipids. Comparable findings were reported by Bhernama et al.,^19^ who showed that decreasing macroalgal particle size to about 0.10 mm significantly increased lipid recovery (up to a 6-fold improvement) when using methanol-hexane solvents. Similarly, AlMohamadi et al.^20^ optimized Soxhlet extraction of a marine macroalgae consortium and identified an optimal particle size of 0.16 mm, which yielded 12.76% lipids, substantially higher than coarser fractions. These results collectively support that particle-size optimization improves extraction efficiency and that mechanical size reduction prior to solvent extraction is an effective pretreatment strategy for *Ulva lactuca* biodiesel production. However, the effect of particle size reduction is primarily associated with increased surface area and enhanced solvent penetration and does not necessarily imply complete disruption of the algal cell wall. This behavior is consistent with intraparticle diffusion extraction reported in other macroalgae species, indicating that *U. lactuca* exhibits similar equilibrium characteristics rather than showing distinct deviations. Nevertheless, although particle size reduction improved lipid extraction efficiency, excessive grinding may increase process energy consumption. Therefore, future studies should evaluate the trade-off between lipid recovery and grinding energy demand to assess the practical feasibility of biodiesel production.

### 3.2 Effect of Extraction Temperature on Lipid Yield

Figure 3 illustrates the influence of extraction temperature on lipid yield from *Ulva lactuca* using Soxhlet extraction with a methanol-hexane (2:1 v/v) solvent system. Lipid yield increased progressively with extraction time at all temperatures, reflecting a time-dependent diffusion process. The extraction performed at higher temperatures, such as 85 and 95 °C, consistently produced greater yields than at 75 °C. Lipid recovery reached approximately 5.04% at 75 °C, 9.48% at 85 °C, and 11.93% at 95 °C. This trend indicates that elevating temperature enhances solvent diffusivity and solute solubility, thereby accelerating the rate of mass transfer between the solvent and algal matrix. However, the yield increment began to level off beyond 300 min, suggesting that most extractable lipids had been recovered and equilibrium was approached.

**Figure 3:**
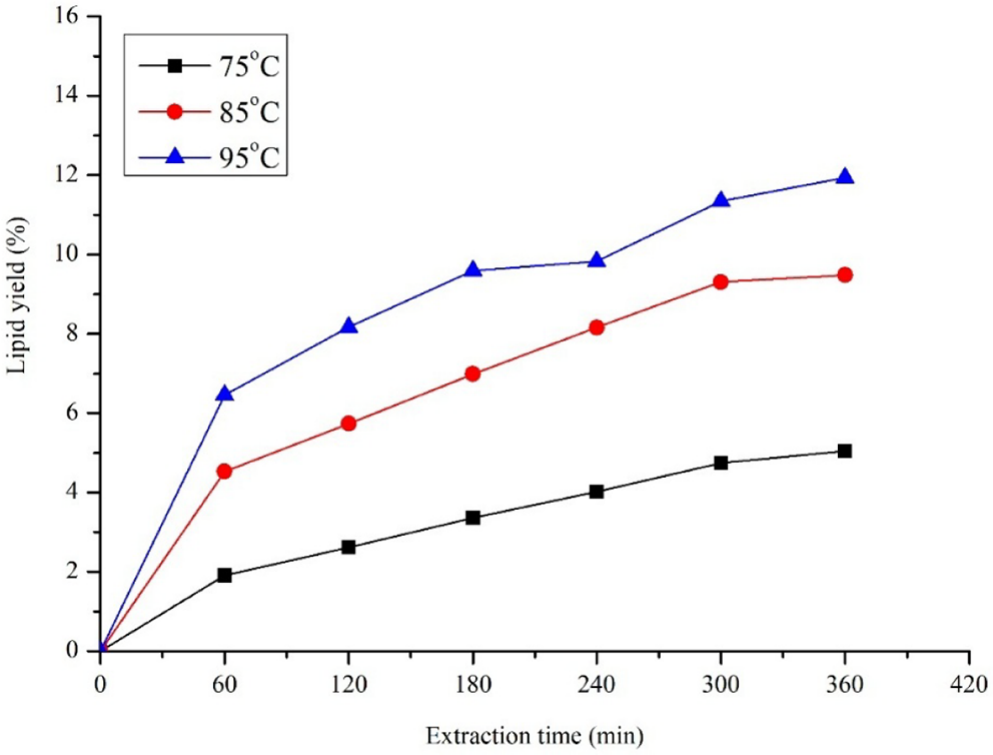
Effect of extraction temperature on lipid yield from *Ulva lactuca* during Soxhlet extraction.

The positive correlation between extraction temperature and lipid yield is attributed to enhanced solvent volatility and reflux at higher temperatures, which improves contact efficiency while lowering the solvent’s viscosity and surface tension.^21^ These thermal effects enable the solvent to penetrate the algal cell matrix more rapidly and dissolve lipids more efficiently, thereby accelerating lipid release. Empirical studies support this trend, where an optimization study on *Ulva lactuca* found the highest lipid recovery at roughly 70 °C under Soxhlet extraction conditions, yielding about 10% of lipids from the biomass.^22^ In general, moderate increases in temperature lead to sharply higher lipid yields due to improved solvent diffusivity. However, excessively high temperatures can be detrimental. Previous study reported that beyond 70 °C, the lipid yield actually decreased because of oxidative degradation of thermolabile unsaturated fatty acids.^23^

The plateau in yield observed during the later stages of extraction at 95 °C suggests that most readily soluble lipids had already diffused into the solvent, while the remaining fractions were either less accessible or prone to thermal breakdown. This behavior can be attributed to the increased solvent evaporation and condensation rate at elevated temperatures, resulting in more frequent Soxhlet reflux cycles and enhanced solvent renewal. Consequently, the *Ulva Lactuca* biomass is exposed to fresh solvent more frequently, which improves lipid extraction efficiency. At the same time, elevated temperatures promote partial degradation of thermolabile unsaturated fatty acids, indicating a trade-off between increased extraction efficiency and preservation of lipid quality. Overall, the present results confirm that temperature is a critical factor in accelerating lipid extraction from algae by enhancing solvent–solute interactions and mass-transfer rates. However, careful temperature control is necessary to prevent degradation of the lipid profile.

### 3.3 Extraction Kinetics Modeling

In this work, the extraction data were fitted to a second-order kinetic model, and the parameters—rate constant (*k*_s_), equilibrium concentration (C_s_), and initial extraction rate (h_s_) were calculated along with the coefficient of determination (*R* ^2^). The fitted model curves are presented in Figure 4a and b, and the corresponding parameters are summarized in Table 1. The *R* ^2^ values for both particle-size groups exceeded 0.95, indicating that the second-order model accurately represents the extraction kinetics. All kinetic parameters increased with temperature, highlighting the strong influence of thermal energy on solvent–solute interaction and overall extraction efficiency under Soxhlet conditions.^24^

**Table 1:** Kinetic Parameters of the Two Sample Types at Different Temperatures

Sample Type	T (°C)	R^2^	*k*_s_ (g/mg/min)	*h*_s_ (%/min)	*C*_s_ (%)
Unground	95	0.9858	7.70 × 10^–05^	0.8251	10.35
Unground	85	0.9951	6.92 × 10^–05^	0.4925	8.44
Unground	75	0.9936	6.13 × 10^–05^	0.2738	6.68
Ground	95	0.9841	7.84 × 10^–05^	1.6383	14.46
Ground	85	0.9764	5.62 × 10^–05^	0.954	13.03
Ground	75	0.9547	5.32 × 10^–05^	0.3519	8.13

**Figure 4:**
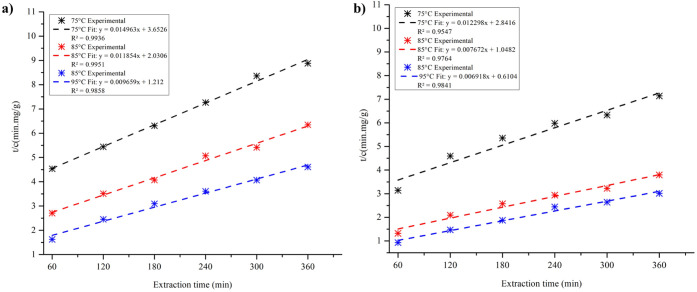
Second-order kinetic model for lipid extraction using Soxhlet at different temperatures and times. (a) unground samples and (b) ground samples.

Comparing the rate constants revealed that the *k*_s_ of the ground sample (7.84 × 10^–5^ g mg^–1^ min^–1^) in Figure 4b was slightly higher than that of the unground sample (7.70 × 10^–5^ g mg^–1^ min^–1^) in Figure 4a at 95 °C. Conversely, at 75 and 85 °C, the unground samples exhibited marginally higher *k*_s_ values, indicating that structural disruption through grinding alone does not provide a distinct kinetic advantage under lower or moderate thermal conditions. This is because, in the Soxhlet system, higher heating intensity primarily accelerates the solvent evaporation–condensation cycles rather than directly heating the sample. At lower temperatures, the slower reflux frequency limits solvent renewal and contact with the biomass, keeping the process diffusion-limited despite increased surface area from grinding. In contrast, higher heating enhances the number of extraction cycles per unit time, ensuring more frequent washing with fresh solvent and greater mass-transfer efficiency. Consequently, the kinetic advantage of ground samples becomes evident only when sufficient thermal energy sustains rapid solvent circulation within the Soxhlet extractor.

The enhancement in the extraction rate becomes more pronounced at elevated temperatures, implying that the effect of particle size on lipid diffusion is temperature-dependent. The equilibrium concentration (*C*_s_) of the ground samples was markedly higher than that of the unground samples at all temperatures, demonstrating that fragmentation of the cellular matrix substantially increases the specific surface area available for solvent penetration and contact with intracellular lipids. This improved accessibility results in higher lipid release potential from the ground biomass.^25,26^ Similarly, the initial extraction rate (*h*_s_) of the ground samples exceeded that of the unground group across all conditions, indicating that mechanical size reduction accelerates the early-stage diffusion and migration of lipids into the solvent phase, thereby improving the overall extraction rate.

### 3.4 Extraction Thermodynamic Modeling

The thermodynamic parameters of lipid extraction from *Ulva lactuca* were evaluated using Arrhenius and Eyring models, including activation energy (*E*_*a*_), enthalpy change (Δ*H*), entropy change (ΔS), and Gibbs free energy change (Δ*G*). The results are summarized in Table 2, and the corresponding linear relationships are presented in Figure 5.

**Table 2:** Thermodynamic Parameters of the Two Sample Types

Sample Type	*E*_a_ (kJ/mol)	Δ*H* (kJ/mol)	*ΔS* (kJ/mol·K)	*ΔG* (kJ/mol)	*A* (g/mg/min)
Ground	20.53	17.56	–0.278	117.12	6113.61
Unground	12.16	9.19	–0.103	46.04	409.94

**Figure 5:**
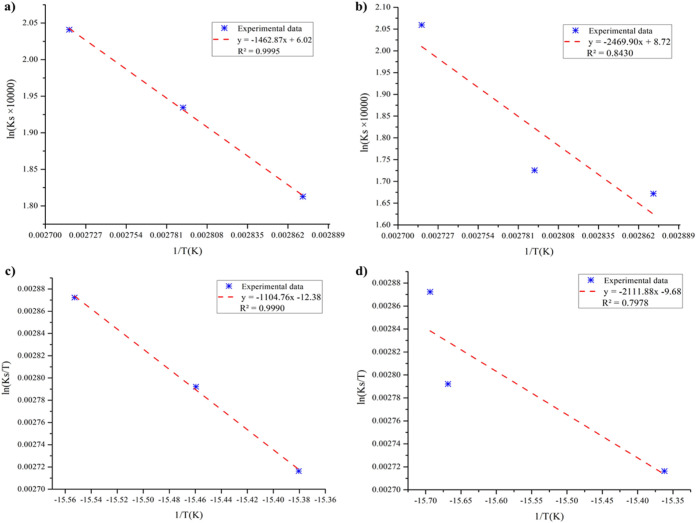
Arrhenius and Eyring plots for lipid extraction kinetics of *Ulva lactuca* using the Soxhlet method. (a) Arrhenius plot of ln­(*k*_s_) versus 1/T for the unground sample, (b) Arrhenius plot of ln­(*k*_s_) versus 1/T for the ground sample, (c) Eyring plot of ln­(*k*_s_ */ T*) versus 1/T for the unground sample, and (d) Eyring plot of ln­(*k*_s_ */ T*) versus 1/T for the ground sample.

The *E*_a_ for the ground sample (20.53 kJ/mol) was higher than that of the unground sample (12.16 kJ/mol). The relatively low activation energy values obtained in this study suggest that Soxhlet extraction process is governed primarily by apparent diffusion-controlled mass transfer rather than by intrinsic chemical reactions. The slightly higher *E*_a_ observed for the ground sample may be associated with differences in solvent penetration and transport resistance within the biomass matrix. Similar low activation energy values have been reported in diffusion-limited extraction systems involving macroalgal biomass.^27,28^

Similarly, the *ΔH* was higher for the ground sample (17.56 kJ/mol) compared to the unground sample (9.19 kJ/mol), confirming that the extraction process is endothermic and temperature dependent. The positive *ΔH* values indicate that thermal energy supports lipid extraction by enhancing solvent diffusivity and mass transfer within the biomass matrix. The Eyring plots shown in Figure 5c and d further support the temperature dependence of the extraction process. The *ΔS* was negative for both samples, with the ground sample showing a more negative value (−0.278 kJ/mol·K) than the unground sample (−0.103 kJ/mol·K), indicating reduced randomness during extraction. Similar thermodynamic behavior has been reported in diffusion-controlled lipid extraction systems.^29,30^

The *ΔG* values were positive for both samples, with a significantly higher value observed for the ground sample (117.12 kJ/mol) compared to the unground sample (46.04 kJ/mol). This indicates that the extraction process is nonspontaneous under the studied conditions and requires external thermal energy input. The thermodynamic results collectively suggest that lipid extraction from *Ulva lactuca* under Soxhlet conditions is predominantly governed by apparent diffusion-controlled transport behavior, likely involving intraparticle diffusion.^31,32^ However, the present study did not experimentally distinguish between bulk diffusion and intraparticle diffusion mechanisms. Therefore, further investigation is required to confirm the dominant mass transfer pathway during extraction.

#### 3.4.1 Functional Group Identification

In the FTIR spectra of this study, as shown in Figure 6, the two absorption peaks at 2946.51 cm^–1^ and 2837.38 cm^–1^ correspond to the stretching vibrations of the −CH_2_– and −CH_3_ groups, indicating the possible presence of long-chain saturated fatty acids.^33,34^ Similar peaks appearing in the 2944–2854 cm^–1^ region have been widely reported in natural lipids. This region is recognized as the characteristic infrared absorption range of fatty acids and their ester compounds, and is commonly used to characterize the main chain structure of natural lipids.^22,40^ A strong absorption peak was observed at 1648.44 cm^–1^. Previous studies have reported same peaks and confirming their structural affiliation to esters or carboxylic acids. It is also noted that while the C=O absorption peak of ester groups typically occurs at 1740–1720 cm^–1^ under standard conditions, it can be red-shifted to 1680–1640 cm^–1^.^35^ The presence of this absorption peak thus provides strong evidence for ester compounds in the sample. Two moderate-intensity absorption peaks were observed at 1108.53 cm^–1^ and 1016.63 cm^–1^. Also, similar peaks within the 1300–1000 cm^–1^ region have been explicitly attributed to C–O stretching vibrations in ester structures. Together with the characteristic absorptions in the C–H (2946–2837 cm^–1^) and C=O (1648.44 cm^–1^) regions, the C–O signals in this region further support the presence of ester compounds.^41,42^

**Figure 6:**
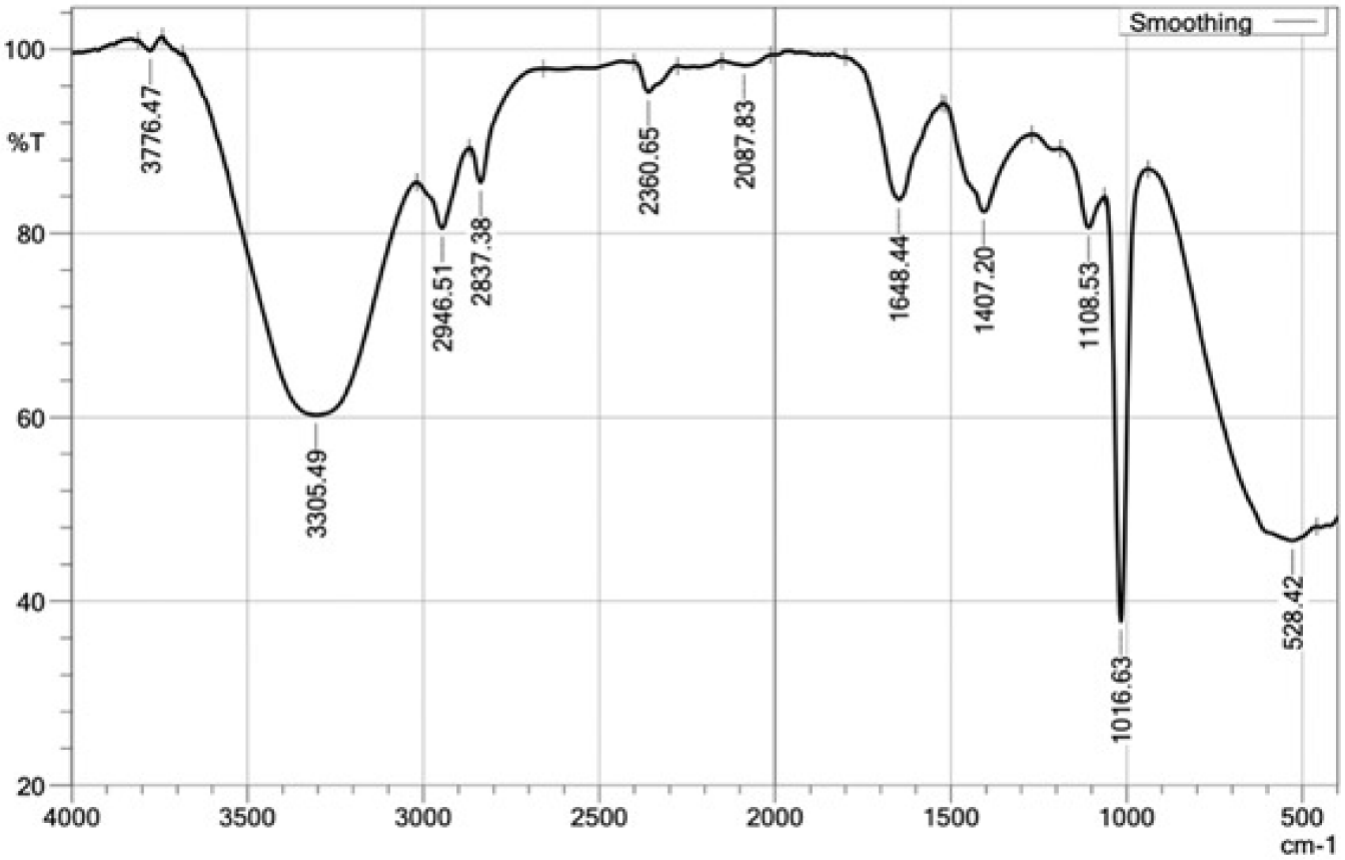
FTIR spectrum of lipid extract obtained from ground *Ulva lactuca* biomass (50–250 μm) using Soxhlet extraction at 95 °C for 360 min.

Additionally, some minor peaks were observed. The absorption peaks at 3776.47 cm^–1^ and 3305.49 cm^–1^ are attributed to stretching vibrations of free hydroxyl groups (−OH), indicating the presence of free fatty acids in the sample.^36^ This observation is consistent with the relatively high acid value obtained, suggesting that the extracted lipid is not purely composed of triglycerides. The presence of free fatty acids may result from lipolysis during biomass drying or storage, as well as from hydrolysis of lipids promoted by elevated extraction temperatures. The peaks at 2360.65 cm^–1^ and 2087.83 cm^–1^ are typically associated with CO_2_ interference, which are background signals with minimal relevance to lipid structure.^37^

### 3.5 Physicochemical Properties

The physicochemical properties of extracted lipids from *Ulva lactuca* are revealed in Table 3. The results were evaluated against the ASTM D6751 and EN 14214 biodiesel standards, with observed deviations analyzed and corresponding improvement recommendations proposed.

**Table 3:** Physicochemical Properties of Extracted Lipids from *Ulva Lactuca*

Physicochemical Properties	Units	ASTM D6751	EN14214	*Ulva lactuca* lipids
Saponification value	mg KOH/g	<370	-	132.25 ± 1.45
Acid value	mg KOH/g	<0.5	<0.5	19.16 ± 0.79
FFA	%	-	-	9.58 ± 0.39
Iodine value	g I_2_/100g	-	>120	56.01 ± 0.95
Kinematic viscosity at 40 °C	mm^2^ /s	1.9–6.0	3.5–5.0	12.1±0.24
Density at 15 °C	kg/m^3^	880	860–900	941.0 ± 8.0

#### 3.5.1 Acid Value

The acid value of the extracted lipid was 19.16 mg KOH g ^–1^ (±4.1%), which is higher than the range typically reported for *Ulva lactuca* lipids (12.00–15.16 mg KOH g^–1^).^38,42^ This elevated value indicates a high concentration of free fatty acids (FFAs), suggesting that partial hydrolysis of triglycerides occurred during or prior to extraction.^43^ The elevated acid value may be attributed to several factors, including environmental conditions such as salinity, temperature, and microbial activity. In addition, postharvest handling factors such as storage duration, exposure to oxygen, and residual moisture can promote lipid hydrolysis. The absence of pretreatment, such as drying optimization or acid esterification prior to transesterification, may have further contributed to the accumulation of FFAs.^39,40^ The high FFA content (>9%) exceeds the typical threshold for base-catalyzed transesterification and may result in soap formation, reduced ester yield, and difficulties in product separation.

#### 3.5.2 Iodine Value

The iodine value (IV) of the extracted lipid averaged 56.01 g I_2_/100 g (±1.69%), which is lower than the range typically reported for *Ulva lactuca* (65.1–97.2 g I_2_/100 g).^47–49^ This indicates a lower degree of unsaturation, suggesting a higher proportion of saturated fatty acids in the extracted lipid fraction.

The variation in iodine value may be attributed to a combination of environmental conditions, extraction methodology, and thermal effects. Environmental factors such as high water temperature, light intensity, salinity, and low nutrient availability can influence fatty acid biosynthesis. These conditions promote the formation of saturated fatty acids and reduce lipid unsaturation.^43^ In addition, solvent selectivity during extraction may limit the recovery of certain unsaturated lipid fractions. While methanol is effective for polar lipids, it may have limited solubility for certain nonpolar unsaturated triglycerides, which could result in an underestimation of the iodine value.^44^ Prolonged exposure to elevated temperatures during extraction (6 h at 95 °C) and subsequent reflux (75 °C) may induce thermal degradation of unsaturated fatty acids. These conditions can reduce the number of reactive double bonds, thereby lowering the measured iodine value.^45^

While a reduced iodine value may improve oxidative stability and storage performance, it can negatively affect cold-flow properties due to the higher proportion of saturated fatty acids. Therefore, optimization of extraction conditions, including solvent selection, temperature control, and extraction duration, is necessary to balance lipid recovery and fuel properties.

#### 3.5.3 Saponification Value

The saponification value (SV) of the extracted lipid averaged 132.25 mg KOH g^–1^ (±1.1%), demonstrating good analytical reproducibility and consistency across measurements. This value complies with the requirements of ASTM D6751 for biodiesel feedstocks, indicating that the lipid possesses acceptable ester content for biodiesel production. However, the SV obtained in this study is substantially lower than values previously reported for *Ulva lactuca* lipids, such as 213.4 ± 1.6 mg KOH g^–1^ reported by Binhweel et al.,^46^ highlighting notable differences in lipid composition between studies. The relatively low saponification value may be attributed to the predominance of long-chain fatty acids and higher molecular weight lipid components, which contain fewer ester linkages per unit mass and therefore require less potassium hydroxide for saponification.^47^ This interpretation is supported by the fatty acid profile obtained from GC-FID analysis.

From a biodiesel performance perspective, a lower saponification value may have both advantageous and limiting implications. Longer-chain and saturated fatty acids generally enhance the cetane number and oxidative stability of the resulting biodiesel, they may adversely affect cold-flow properties, such as cloud point and pour point. Therefore, the relatively low SV observed in this study reflects a lipid composition that may favor combustion efficiency and storage stability, albeit with potential trade-offs in low-temperature operability. These findings underscore the importance of integrating saponification value analysis with detailed fatty acid profiling when assessing the suitability of algal lipids as biofuel feedstocks.

#### 3.5.4 Density

The density of the extracted lipid was determined to be 0.941 g mL^–1^ (±0.85%), which exceeds the ASTM D6751 specified range for biodiesel (0.86–0.90 g mL^–1^) and is marginally higher than the value reported for *Ulva lactuca* lipids by Binhweel et al.,^22^ who reported a density of 0.889 g mL^–1^. Density is a critical fuel property as it directly influences mass-based fuel injection, spray characteristics, and combustion efficiency in diesel engines. Deviations from standard density ranges may, therefore, affect engine performance and emission behavior if not adequately controlled.

The elevated density observed in this study can be attributed to the presence of residual polar and high-molecular-weight impurities coextracted with the lipid fraction. Macroalgal biomass is known to contain substantial amounts of phospholipids, glycolipids, proteins, chlorophyll, and other pigments, which possess higher densities than neutral triglycerides.^48^ Incomplete separation of these components during lipid extraction and fractionation can increase the overall density of the recovered lipids. Additionally, incomplete removal of methanol or other polar solvents used during extraction and fractionation may further contribute to the increased density, as residual solvent molecules can remain entrapped within the lipid matrix.

Extraction conditions, including solvent polarity, temperature, and extraction duration, play a significant role in determining lipid purity and density. The use of methanol, while effective for disrupting algal cell walls and extracting polar lipid fractions, may promote coextraction of nonlipid compounds, thereby increasing the density of the recovered lipid phase.^49^ Prolonged extraction or insufficient postextraction solvent evaporation may exacerbate this effect, leading to higher measured density values.^50^

Although the measured density exceeds ASTM D6751 specifications, this result does not preclude the suitability of the lipid as a biodiesel feedstock. Instead, it highlights the necessity of additional purification or fractionation steps prior to transesterification. Techniques such as solvent washing, degumming, adsorption using silica or activated carbon, and vacuum-assisted solvent removal could effectively reduce impurity levels and bring the density closer to that of standard biodiesel requirements. Implementing such purification strategies is expected to improve transesterification efficiency, enhance fuel quality, and ensure better compliance with biodiesel standards.

#### 3.5.5 Kinematic Viscosity

The kinematic viscosity of the extracted lipid was measured at 12.10 mm^2^ s^–1^ at 40 °C, which substantially exceeds the ASTM D6751 specified range for biodiesel (1.9–6.0 mm^2^ s^–1^). Kinematic viscosity is a critical fuel property that governs atomization quality, fuel spray penetration, and air-fuel mixing during combustion. Excessively high viscosity can lead to poor atomization, incomplete combustion, increased injector fouling, and elevated exhaust emissions. Consequently, the viscosity value obtained in this study indicates that the lipid feedstock, in its raw form, is not directly suitable for use as biodiesel and requires transesterification or further processing to reduce viscosity and achieve compliance with fuel standards.^51^

This higher viscosity is expected, as the value corresponds to the raw lipid extract rather than the final biodiesel product, where triglycerides and long-chain fatty acids contribute to increased resistance to flow. Upon transesterification to fatty acid methyl esters (FAMEs), the viscosity is anticipated to decrease significantly, falling within the acceptable biodiesel range. Previous studies have demonstrated that feedstocks with high saturated fatty acid content tend to exhibit increased viscosity and inferior cold-flow properties, including elevated cloud point, pour point, and cold filter plugging point (CFPP), which collectively impair fuel flowability at lower temperatures.^52^ Saturated fatty acids possess linear molecular structures that promote stronger intermolecular interactions and tighter molecular packing, resulting in increased resistance to flow and, consequently, higher viscosity.

GC-FID analysis conducted in this study confirmed the predominance of saturated fatty acids in the *U. lactuca* lipid extract, particularly palmitic acid (C16:0) and stearic acid (C18:0). These long-chain saturated fatty acids are known to significantly increase the viscosity of lipid feedstocks due to their high melting points and enhanced van der Waals interactions.^53^ The abundance of these components, therefore, provides strong compositional evidence supporting the high kinematic viscosity measured.

From a processing perspective, transesterification is essential to convert triglycerides into fatty acid methyl esters (FAMEs), which exhibit substantially lower viscosities and improved flow characteristics. Alternatively, blending with lower-viscosity fuels, winterization, or the use of cold-flow improvers may further mitigate viscosity-related limitations. However, transesterification remains the most effective and widely adopted approach for viscosity reduction in biodiesel production.

## 4 Conclusions

This study provides a comprehensive assessment of lipid extraction from *Ulva lactuca* using Soxhlet methodology, integrating kinetic and thermodynamic modeling with physicochemical and structural characterization. Fine grinding and elevated temperatures up to 95 °C significantly improved lipid yield, reaching 11.93% due to enhanced mass transfer and solvent interaction. The extraction followed a second-order kinetic model with high *R* ^2^ values (>0.95). Thermodynamic analysis confirmed that the process is endothermic and nonspontaneous, highlighting its strong temperature dependence. This indicates that Soxhlet extraction is not inherently inefficient, but rather that extraction performance is governed by thermal conditions, requiring adequate temperature to overcome mass transfer resistance and achieve effective lipid recovery. FTIR analysis confirmed the presence of lipid-associated functional groups, while GC-FID profiling revealed a high proportion of saturated fatty acids. However, the extracted lipids exceeded acceptable limits for key physicochemical properties when benchmarked against ASTM D6751 and EN 14214 standards, with an acid value of 19.16 mg KOH/g (limit: < 0.5 mg KOH/g), kinematic viscosity of 12.1 mm^2^ /s (limit: 1.9–6.0 mm^2^ /s), and density of 941.0 kg/m^3^ (limit: 860–900 kg/m^3^). These deviations are attributed to high FFA content, saturation, and possible thermal degradation. Nonetheless, the low iodine value and high oxidative stability suggest favorable storage potential. Overall, this work deepens understanding of the extraction behavior and macromolecular properties of marine algal lipids. While additional purification and pretreatment steps are required to meet biodiesel quality standards, *Ulva lactuca* remains a viable and sustainable lipid source for third-generation biodiesel. The mechanistic insights gained from this study lay the groundwork for future optimization and scaling of algae-based biofuel systems.

## Data Availability

Data sets and analyses from this study can be provided by the corresponding author upon reasonable request. All data in this study are presented in the manuscript.
